# MicroRNA-target interactions in neurodegenerative diseases

**DOI:** 10.1186/2193-1801-4-S1-L1

**Published:** 2015-06-12

**Authors:** Hermona Soreq

**Affiliations:** The Hebrew University of Jerusalem, Israel

**Keywords:** Alzheimer’s disease, microRNAs, pseudogenes

MicroRNAs (miRNAs) are short, 22-25 nucleotide long transcripts that may suppress entire signaling pathways by interacting with the 3’-untranslated region (3’-UTR) of coding mRNA targets, interrupting translation and inducing degradation of these targets. The long 3’-UTRs of brain transcripts compared to other tissues predict important roles for brain miRNAs. Supporting this notion, we found that brain miRNAs co-evolved with their target transcripts, that non-coding pseudogenes with miRNA recognition elements compete with brain coding mRNAs on their miRNA interactions, and that Single Nucleotide Polymorphisms (SNPs) on such pseudogenes are enriched in mental diseases including autism and schizophrenia, but not Alzheimer’s disease (AD). Focusing on evolutionarily conserved and primate-specific miRNA controllers of cholinergic signaling (‘CholinomiRs’), we find modified CholinomiR levels in the brain and/or nucleated blood cells of patients with AD and Parkinson’s disease, with treatment-related differences in their levels and prominent impact on the cognitive and anti-inflammatory consequences of cholinergic signals. Examples include the acetylcholinesterase (AChE)-targeted evolutionarily conserved miR-132, whose levels decline drastically in the AD brain. Furthermore, we found that interruption of AChE mRNA’s interaction with the primate-specific CholinomiR-608 in carriers of a SNP in the AChE’s miR-608 binding site induces domino-like effects that reduce the levels of many other miR-608 targets. Young, healthy carriers of this SNP express 40% higher brain AChE activity than others, potentially affecting the responsiveness to AD’s anti-AChE therapeutics, and show elevated trait anxiety, inflammation and hypertension. Non-coding regions affecting miRNA-target interactions in neurodegenerative brains thus merit special attention.

